# INVASIVE NON-TYPE B *HAEMOPHILUS INFLUENZAE* DISEASE: REPORT OF EIGHT CASES

**DOI:** 10.1590/1984-0462/;2019;37;2;00006

**Published:** 2019-01-07

**Authors:** Mariana Tresoldi das Neves Romaneli, Antonia Teresinha Tresoldi, Ricardo Mendes Pereira, Márcia Teixeira Garcia, Christian Cruz Hofling, Mariângela Ribeiro Resende

**Affiliations:** a Universidade Estadual de Campinas, Campinas, SP, Brazil.

**Keywords:** Ampicillin, Haemophilus influenzae, Child, Ampicilina, Haemophilus influenzae, Criança

## Abstract

**Objective:**

To describe eight cases of invasive non-type b *Haemophilus influenzae* disease in children admitted to Hospital de Clínicas of Universidade Estadual de Campinas.

**Cases description:**

In 2015, there were eight cases of invasive non-type b *H. influenzae* disease. We tested the ampicillin sensitivity and beta-lactamase production of the strains identified and performed the genotyping. Molecular typing was determined by Pulsed-Field Gel Electrophoresis. Four patients were diagnosed with bacteremia; in two cases, *H. influenzae* was detected in the pleural fluid, and two patients had meningitis. Patients with comorbidities represented 37.5% of cases. Except for the strain of one patient - not sent to the reference laboratory -, all were ampicillin-sensitive and non-beta-lactamase-producing. Genotyping identified four non-capsular, one type c, and two type a strains. Molecular typing ruled out nosocomial transmission since all serotypes were distinct regarding genotype.

**Comments:**

The rise in cases of invasive non-type b *H. influenzae* infection was real. There was no nosocomial transmission, and we found no justification for the increase. These data indicate the need for surveillance to correctly diagnose, monitor, and understand the spectrum of non-type b *H. influenzae* disease.

## Abstract (pt)

**Objetivo:**

Descrever oito casos de doença invasiva por *Haemophilus influenzae* não tipo b em crianças internadas no Hospital de Clínicas da Universidade Estadual de Campinas.

**Descrição dos casos:**

Em 2015, ocorreram oito casos de doença invasiva por *H. influenzae* não tipo b. Nas cepas identificadas, testou-se a sensibilidade à ampicilina e a produção de betalactamase, e realizou-se a genotipagem. A tipagem molecular foi feita por *Pulsed Field Gel Electrophoresis*. Em quatro pacientes, o diagnóstico foi de bacteremia; em dois casos, *H. influenzae* foi identificado em líquido pleural, e dois pacientes tiveram meningite. Comorbidades foram encontradas em 37,5% dos pacientes. Com exceção da cepa de um dos pacientes (que não foi enviada ao laboratório de referência), todas eram sensíveis à ampicilina e não produtoras de betalactamase. A genotipagem identificou quatro cepas não capsulares, uma cepa tipo c e duas cepas tipo a. A tipagem molecular descartou a transmissão intra-hospitalar, já que todos os sorotipos eram distintos quanto ao genótipo.

**Comentários:**

O aumento dos casos de infecção invasiva por *H. influenzae* não tipo b foi real. Não houve transmissão intra-hospitalar e não foi encontrada justificativa para o aumento. Esses dados indicam a necessidade de vigilância para diagnosticar corretamente, monitorar e entender o espectro da doença causada por *H. influenzae* não tipo b.

## INTRODUCTION

*Haemophilus influenzae* (Hi) is a Gram-negative coccobacillus regarded as a component of the normal flora of the upper respiratory tract of human beings.^1^ It is classified according to the presence of one among six serologically distinct capsular polysaccharides, called serotypes a, b, c, d, e, and f, identified by agglutination in the presence of a specific serum for each serotype.^2^ Non-encapsulated *Haemophilus influenzae* are known as non-typable (NTHi) since they cannot be sorotyped by conventional agglutination with a determined antiserum.

The incidence of severe Hi infections sharply decreased since the introduction of the vaccine against serotype b.^3^ Although all serotypes can cause diseases, especially in infants and children, NTHi strains have been frequently reported as the source of invasive diseases.^4^

The objective of this work was to describe eight cases of invasive non-type b Hi disease, which occurred in 2015 at the pediatric hospitalization unit of Hospital de Clínicas of Universidade Estadual de Campinas (HC-Unicamp), São Paulo.

## CASES DESCRIPTION

The pediatric hospitalization unit comprises the pediatric ward (48 beds) and the pediatric Intensive Care Unit (ICU) (10 beds), receives clinical and surgical patients, and belongs to the HC-Unicamp, a tertiary and general teaching hospital. The average number of monthly admissions is 170 patients at the ward and 35 at the ICU.

Epidemiological surveillance identified one child with invasive Hi disease in 2013, and two children received the same diagnosis in 2014. However, between March and August of 2015, eight cases of invasive non-type b Hi disease were detected in patients admitted to the pediatric hospitalization unit.

The Hi strains isolated from patients were identified at the Clinical Pathology Laboratory of HC-Unicamp, and subsequently sent to Instituto Adolfo Lutz, in São Paulo, which rechecked the identification, performed a disk diffusion antibiogram, sensitivity tests by minimum inhibitory concentration (MIC), beta-lactamase assay, capsular type assay, genotyping by polymerase chain reaction (PCR) in real time, and molecular typing by Pulsed-Field Gel Electrophoresis (PFGE) in six strains.

Three patients (cases 2, 3, and 6) were diagnosed with bacteremia without focus, despite being in ICU for acute viral bronchiolitis for more than 10 days. In two patients (cases 1 and 4), the bacterium was isolated in a pleural fluid sample present on admission. In one patient (case 5), admitted for adrenal tumor surgery and with symptoms of upper respiratory infection, the bacterium was isolated in the blood culture on the first day of hospitalization. Two patients (cases 7 and 8) were diagnosed with meningitis; case 7 within 48 hours of hospitalization and case 8, five days after admission (Table 1). Both patients with meningitis had a predisposing risk factor: one of them had a history of skull base trauma (with multiple orbital fractures), and the other had a diagnosis of pansinusitis related to giant cervical lymphangioma. The identified comorbidities were asthma (case 1), adrenal tumor (case 5), and giant cervical lymphangioma (case 7), representing 37.5% of patients. Except for case 7, who died, all patients had a favorable outcome.

**Table 1:** Information related to patients and *H. influenzae* strains isolated from each patient.

Case	Date	Age/comorbidity	Admission diagnosis/site of isolation/place of hospitalization	Sensitivity to ampicillin-antibiogram/MIC	Beta-lactamase	Genotyping
1	04/11	8 years/mild asthma	Pneumonia with pleural effusion/blood and pleural fluid/ICU	Sensitive/1 µg/mL	Negative	Type c
2	05/03	7 m/absent	AVB in MV/blood/ICU	Sensitive/0.125 µg/mL	Negative	Non-capsular
3	05/10	1 m/absent	AVB in MV/blood/ICU	Sensitive/0.5 µg/mL	Negative	Non-capsular
4	05/18	5 years and 7 m/absent	Pneumonia with pleural effusion/pleural fluid/W	Resistant/NP	NP	NP
5	05/28	1 year and 9 m/adrenal carcinoma	Viral respiratory disease and preoperative/blood/W	Sensitive/0.25 µg/mL	Negative	Non-capsular
6	07/14	1 m/absent	AVB in MV/blood/ICU	Sensitive/0.5 µg/mL	Negative	Type a
7	08/11	4 years, giant cervical lymphangioma, tracheostomy, and gastrostomy	Pansinusitis, ICH, and bilateral cerebral vasculitis/liquor/ICU	Sensitive/0.25 µg/mL	Negative	Type a
8	08/21	6 years/absent	TBI and multiple orbital fractures/blood and liquor/W	Sensitive/0.125 µg/mL	Negative	Non-capsular

MIC: minimum inhibitory concentration - CLSI criteria of the current year: sensitive (≤1 µg/mL); undetermined (=2 µg/mL); and resistant (≥4 µg/mL); m: months; ICU: Intensive Care Unit; AVB: acute viral bronchiolitis; MV: mechanical ventilation; W: ward; ICH: intracranial hypertension; TBI: traumatic brain injury; NP: not performed.

All children had been vaccinated against type b Hi and taken the appropriate number of doses for their age group, except for two infants with one month of age (cases 3 and 6), as they had not reached the minimum age to receive the vaccine. Regarding the prior use of antibiotics, cases 2, 3, 5, 6, and 8 had no history of antibiotic therapy during the month prior to hospitalization, while cases 1, 4, and 7 had taken, respectively, oral amoxicillin, intravenous crystalline penicillin, and amoxicillin-clavulanate by gastrostomy.

After the identification of the fifth case, the situation was investigated as a possible hospital outbreak; for this reason, the strain isolated from patient number 4, recovered from a pleural fluid sample, was not sent to the reference laboratory. The possibility of nosocomial transmission existed since four cases had been reported in the same month, even though they occurred in patients hospitalized in distinct physical areas (ICU and ward) and with different medical and nursing teams.

**Figure 1:**
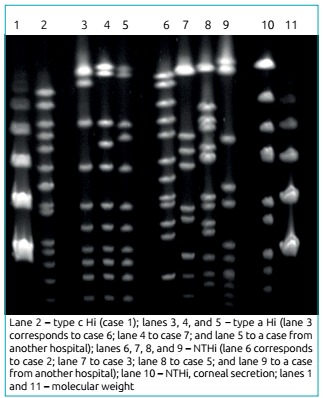
Genomic DNA profile of *H. influenzae* strains isolated from six patients obtained by Pulsed-Field Gel Electrophoresis.

The reference laboratory also conducted a molecular typing by PFGE in strains of six children from the outbreak and strains of two children hospitalized at another hospital in the same city to rule out nosocomial transmission. Figure 1 indicates that type a strains were genetically correlated as they belong to the same capsular type but were not identical. NTHi strains showed high genetic variability, not characterizing a spread of bacteria among patients.

During the investigation, the Epidemiological Surveillance Center guided expanded surveillance, asking for isolated strains in other sterile materials, such as pleural fluid. Procedures in the hospitalization units and microbiology laboratory were reviewed. Blood culture collection did not increase in comparison with the previous three years. Also, sample processing in the laboratory, the automated process (blood culture), and the processing of other materials did not change. Similarly, the incidence of invasive Hi disease did not increase in the neonatology service, which, although located in another building, has the cultures performed in the same laboratory. The Research Ethics Committee of Unicamp approved this study (011/2017) on June 27, 2017.

## DISCUSSION

Nosocomial Hi transmission is possible since the bacteria colonize the upper airway. This colonization in children was already known, but with the introduction of the conjugate pneumococcal vaccine, NTHi colonization has significantly increased among vaccinated children.^5^ ^,^ ^6^ ^,^ ^7^ Nasopharyngeal colonization research performed in Goiânia with 1,192 children showed that 32.1% of them had Hi in the nasopharynx, with 23.3% being non-typable.^8^ Another study described the nosocomial transmission of NTHi strains.^9^ In the present study, this was a non-remote possibility, as the cases occurred in a much larger number than usual and in a short period. However, the tests conducted showed that the infections resulted from two different Hi serotypes (a and c) and, in four cases, from NTHi, all of them distinct from a genotypic point of view, which eliminated the possibility of nosocomial transmission.

Usually, NTHi causes upper airways diseases in children, such as otitis and sinusitis.^10^ Nonetheless, it can lead to invasive diseases, e.g., bacteremia, pneumonia, and meningitis.^2^ ^,^ ^11^ ^,^ ^12^ Some non-type b encapsulated serotypes predominate in distinct geographic locations, such as type a in northern Canada and Israel, ^11^ ^,^ ^13^ ^,^ ^14^ while in other surveillance systems, there is a prevalence of serotype e or f.^2^ ^,^ ^12^ ^,^ ^15^ Infections caused by serotypes c and d are rare, and there is little information about them, with few case reports on the subject.^11^ However, all serotypes can result in invasive diseases, such as bacteremia, meningitis, pneumonia, and, more rarely, septic arthritis.^2^ ^,^ ^11^ ^,^ ^16^ ^,^ ^17^

Langereis et al*.* indicate that a real increase in invasive NTHi diseases is happening, reporting a six times greater incidence in the past two decades in the Netherlands.^4^ On the other hand, among neonates in England, the frequency remains between 2.1 and 4.8/100,000 infants from 2000 to 2013.^18^ Ladhani et al. revealed a slow rise of invasive infections caused by serotypes e and f.^15^ In Brazil, a slight increase in non-type b encapsulated Hi was detected in the first decade after vaccination for type b Hi.^19^ Van Eldere et al. suggest that the growth in incidence reported by several authors can be a combination of factors, such as the identification of the spectrum of the disease, the change in clinical practice (increase in blood culture collection), and the higher sensitivity of laboratory tests with equipment automation.^20^

In Canada, an epidemiological study conducted from 2004 to 2013 showed a sharp increase in the incidence of NTHi, mainly in the extremes of life - children younger than 1 year and adults older than 65 years. The authors suggest that NTHi, type a Hi, and type f Hi are emerging pathogens and should be monitored.^21^ In most of these studies, the higher incidence of NTHi infections in patients with comorbidities could be related to their increased survival. Analyzing the risk factors connected to invasive disease in children, Livorsi et al. associated invasive NTHi diseases with longer hospital stay and higher mortality, when compared to diseases caused by encapsulated Hi. In the same report, 30.7% of patients had comorbidities, with prematurity, asthma, and immunosuppression as the most common.^22^ Similarly to the literature, the cases described in this study manifested as bacteremia, pneumonia, and meningitis. However, the high incidence of invasive NTHi infections in this service had never been reported before, and we found no justification for this increase. Regarding risk factors, the patients were not premature, had little time of hospitalization, and only three of them had comorbidities, differing from cases found in the literature.

Although the strains isolated from patients here reported showed to be sensitive to ampicillin *in vitro*, this resistance is known since the decade of 1970 and is, usually, around 20-30%.^2^ The main mechanism of resistance is beta-lactamase production mediated by plasmids, with identification of the TEM-1 enzyme and, rarely, the ROB-1.^20^ Beta-lactamase-producing strains are called beta-lactamase-positive ampicillin-resistant (BLPAR), and their frequency depends on the region, reaching 30% in Europe and the United States.^23^ ^,^ ^24^ ^,^ ^25^ Some Hi strains that do not express beta-lactamase are resistant to ampicillin and amoxicillin due to changes in their structure (PBP3). Recent studies have shown a higher resistance rate of beta-lactamase-negative ampicillin-resistant (BLNAR) strains, whose frequency ranges from 3.4 to 31%, but this study did not identify them.^23^ ^,^ ^24^ ^,^ ^25^

In conclusion, the increase in cases of invasive non-type b Hi infection was real, which shows the need for continued surveillance to correctly diagnose the infections, monitor, and understand the spectrum of the disease.
